# MRONJ Risk Related to Dental Implants in Osteoporosis Treated With Denosumab: A Systematic Review

**DOI:** 10.1111/odi.70181

**Published:** 2026-01-08

**Authors:** Rebheca Maria Pereira Santos, Giulia Ottaviani, Roberto Di Lenarda, Matteo Biasotto, Katia Rupel

**Affiliations:** ^1^ Department of Medicine Surgery and Health Sciences, University of Trieste Trieste Italy; ^2^ Azienda Sanitaria Universitaria Giuliano Isontina (ASUGI) Trieste Italy; ^3^ Azienda Sanitaria Friuli Occidentale (ASFO) Pordenone Italy

**Keywords:** antiresorptive therapy, denosumab, dental implants, MRONJ, osteoporosis

## Abstract

**Objectives:**

The aim of this systematic review was to assess the impact of dental implants in osteoporotic patients undergoing treatment with denosumab on MRONJ development, considering both implant surgery‐triggered osteonecrosis (ISTO) and implant presence‐triggered osteonecrosis (IPTO).

**Methods:**

An electronic literature search was conducted applying the clinical PICO question, “What is the effect of dental implants on the risk of developing MRONJ in osteoporotic patients undergoing treatment with denosumab?” The full texts of 239 retrieved articles were screened, and 10 studies were included in the quality assessment and data extraction process.

**Results:**

10 articles were selected for data extraction: 6 case series, 2 cohort studies, 1 case control study, and 1 randomized controlled trial. The main findings reported in these studies were evaluated, including factors such as the timing of implant placement, the influence of peri‐implantitis and comorbidities, antiresorptive therapy, and the cases of MRONJ.

**Conclusions:**

The current literature shows high heterogeneity, small sample sizes, and a lack of standardization, making it difficult to draw definitive conclusions. However, despite MRONJ being relatively rare, important factors were identified that could influence outcomes, such as transitioning from bisphosphonates to denosumab therapy, timing of implant placement, and the presence of peri‐implantitis.

## 1 Introduction

Implantology has been developing rapidly in recent years and since the pioneering studies by Branemark in the 60's until the 21st century, it has been consolidating itself as one of the main alternatives for the treatment of oral, esthetic and functional rehabilitation in partially or totally edentulous arches with great predictability of results (Buser et al. 2017). With the increase in life expectancy of the population, especially in developed countries, the need for rehabilitative treatments increases sharply in the older age group of the population, which, even with a drop in edentulism rates compared to previous years, still concentrates a greater number of tooth losses (Muller et al. 2007; Sendyk et al. 2017; Slade et al. 2014). Most of these aging patients have a variety of comorbidities, which can interfere with the surgery and the outcome of the treatment. Among others, osteoporosis, cancer and rheumatic diseases often lead patients to the chronic and prolonged use of specific medications that may have a marked impact on the results of implant therapies, both immediately and in the long term (Carlos et al. 2024; He et al. 2019; Ouanounou et al. 2016).

Among the systemic alterations commonly found in this age group are the diseases that affect bone metabolism. Osteoporosis, one of the diseases with the highest incidence in the population, which can become evident from 50 to 60 years of age, affecting mainly postmenopausal women, is characterized by a decrease in bone mass, presenting, according to the parameters of the World Health Organization, with reduction in Bone Mineral Density (BMD) by up to 2.5 below the standard value, which leads to microarchitectural deterioration of bone tissue with increased bone fragility and increased risk of fractures by up to 40%, leading to several complications and increased morbidity and mortality (Kanis et al. 2013; Kerschan‐Schindl 2016; NIH Consensus Development Panel on Osteoporosis Prevention, Diagnosis, and Therapy 2001). The medications of choice in the treatment of many skeletal disorders are those that have an antiresorptive action. Bisphosphonates (BPs) and Denosumab (DEN) are currently the most used in therapies that aim to inhibit the loss of bone mass, acting directly on osteoclasts or preventing their formation and function (Cortet et al. 2024; Diab and Watts 2024; Elahmer et al. 2024).

The use of such drugs can lead to side effects that can affect treatment and the patient's quality of life. In dentistry and in the specific case of implant dentistry, the effects of antiresorptive agents can have a decisive impact, both in the short and long term. Such effects depend on a series of factors such as dose, route of administration, and duration of treatment and range from possible interferences in the osseointegration process to the potential appearance of necrotic lesions in the jaws of patients that can lead to a state of morbidity, pain, and discomfort, known as Medication‐Related Osteonecrosis of the Jaws (MRONJ) (Bedogni et al. 2024; Ruggiero et al. 2022; Stavropoulos et al. 2018).

Over the past years, several systematic reviews have been published on the role of BPs in implant osseointegration and the potential role of implants in the genesis of MRONJ (Pishan et al. 2024; Sulaiman et al. 2023), leading to the formulation of clear clinical indications and guidelines. In contrast, there is still a lack of consensus regarding the impact of DEN in the planning and maintenance of implant rehabilitations, both with regard to the risk of MRONJ and implant failure. In fact, DEN is a monoclonal antibody used both in metabolic and in neoplastic bone metabolism disorders that inhibits receptor activator of NF‐κB ligand (RANKL) (Lacey et al. 2012). It has a half‐life of approximately 1 month and when discontinued, the effects of therapy are markedly reduced, as unlike BPs, it is not deposited in the bone. Its therapeutic schedule in the treatment of osteoporosis allows oral surgical interventions to be performed with relatively low risk of developing MRONJ without discontinuation of the therapy (Bedogni et al. 2024; Ruggiero et al. 2022), but its impact on implant therapy remains underexplored. Therefore, the aim of this systematic review of current literature is to assess the impact of dental implants in osteoporotic patients undergoing treatment with DEN on MRONJ development, considering both implant surgery‐triggered osteonecrosis (ISTO) and implant presence‐triggered osteonecrosis (IPTO).

## 2 Materials and Methods

### 2.1 Protocol

A systematic review was conducted in accordance with the Preferred Reporting Items for Systematic Reviews and Meta‐Analyses (PRISMA) guidelines 2020 statement (Page et al. 2021) and registered on PROSPERO (International Prospective Register of Systematic Reviews), under the code CRD42017067170.

### 2.2 Pico Question

The PICO question was formulated as follows:

- P (Population): Patients with osteoporosis undergoing treatment with DEN.
- I (Intervention): Dental implants
- C (Comparison): Patients who did not develop MRONJ
- O (Outcome): Incidence of MRONJ

As secondary outcomes, we sought to extract more specific information from the studies, including demographic and anamnestic characteristics of patients, timing of implant placement, implant failure, the stage of MRONJ according to AAOMS criteria, treatment modalities, doses of DEN until the onset of osteonecrosis, and the resolution of the condition (whether healed or not).

### 2.3 Search Strategy

A literature search was conducted (last search in October 2025) in two electronic databases, PubMed (Medline) and Scopus (Elsevier), with no restriction regarding the year of publication and with language restriction to only English. Boolean operators were used with Medical Subject Headings (MeSH) terms with the following string: (“osteoporosis”[Mesh] OR “postmenopausal osteoporosis”[Mesh] OR “denosumab”[All fields]) AND (“dental implants”[All fields] OR “implant*”[All fields] OR (“dental”[All Fields] AND “implants”[All Fields])) AND (“Bisphosphonate Related Osteonecrosis of the Jaws”[Mesh] OR “Medication Related osteonecrosis of the Jaws”[All fields] OR (“osteonecrosis”[All fields] AND “jaws”[All fields]) OR “MRONJ” [All Fields] OR “BRONJ” [All Fields]).

A manual search was also conducted using internet browsers, articles referenced in systematic reviews found during the previous research, and relevant publications to identify any additional studies that may have been missed in the primary search. The same selection criteria were then applied to include the relevant articles for data extraction.

### 2.4 Eligibility Criteria

After duplicates removal, the yielded titles and abstracts of potentially relevant articles were screened by two independent reviewers (RS and KR) according to the following inclusion and exclusion criteria:

Inclusion criteria:

- Patients with osteoporosis on DEN therapy who have undergone dental implants before, during, or after DEN therapy.
- Human studies.
- English language.

Exclusion criteria:

- Studies describing subjects with previous head/neck radiotherapy or oncologic patients in the same group of osteoporotic patients under DEN therapy.
- Animal studies.
- Case series (with fewer than 4 patients).
- Studies with missing/unclearly presented data.

### 2.5 Data Extraction and Reporting

Literature search and data extraction were performed by two independent researchers (RS and KR) and then merged to identify possible disagreements that were discussed with a third party (GO). Data to extract were defined prior to commencement of the study and were documented on an Excel sheet. The following data were determined: gender, age, comorbidity, smoking history, drug holiday, time of implant placement (IPTO/ISTO), other medications used during DEN therapy, dose and mean time of DEN therapy, dental implant insertion, region of dental implant insertion, dental implant survival and loss, presence of peri‐implantitis, previous antiresorptive therapy, development of MRONJ, trigger of MRONJ, localization and timing until MRONJ manifestation, MRONJ therapy and resolution.

Data were organized into tables, including the authors' names, year of publication, and study design. Because of the high degree of heterogeneity among the studies, the lack of standardization or clear distinction between the groups, and the absence of certain key data, a meta‐analysis was not feasible. As a result, the findings have been synthesized narratively, with a focus on the individual results of each study, identifying common trends and variations.

### 2.6 Quality and Risk of Bias Assessment

The quality assessment of the included studies was conducted using different tools to evaluate the risk of bias because of the heterogeneity of the study designs. The ROBINS‐I tool (Cochrane) was used for randomized controlled trials (RCTs) (Sterne et al. 2016), the Newcastle‐Ottawa Scale (NOS) was applied to retrospective cohort studies (Wells et al. 2013), and the JBI Critical Appraisal Checklist was employed for case series and case control (Munn et al. 2020) studies. Each study received a score on the basis of the selection of participants, comparability between groups (where applicable), follow‐up, and outcome assessment. All evaluations were performed by two independent researchers (RS and KR) and then merged to identify possible disagreements that were discussed with a third party (MB) and carefully reviewed to ensure reliability and accuracy. The results of all quality assessments were synthesized into a summary table.

## 3 Results

### 3.1 Study Selection

**FIGURE 1:**
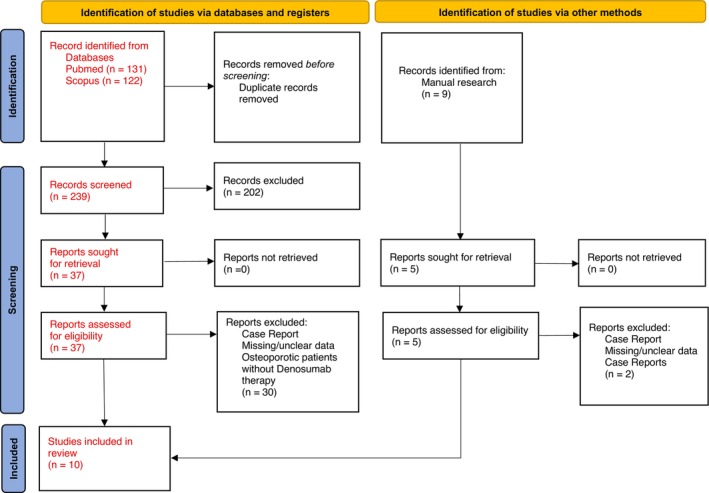
PRISMA flow diagram of the screening and selection process.

The initial search identified a total of 252 articles, 131 articles in the PubMed database and 122 in Scopus (Elsevier). 37 potentially relevant articles remained and were fully assessed and evaluated considering the eligibility criteria. 19 manuscripts were initially deemed relevant, but further exclusions were made because of study types, missing data, or the lack of a clear distinction between groups of osteoporotic patients and other diseases considered as exclusion criteria. This process resulted in a total of 7 articles being included in the review. Simultaneously, a manual electronic search was conducted using articles referenced in systematic reviews and other relevant studies across various publication sites and databases. This additional search applied the same criteria as the primary search, resulting in the inclusion of 3 more articles. Therefore, 10 articles were included in the quality and risk of bias assessment phase. The flow chart for study selection adapted from PRISMA statement is shown in Figure 1.

### 3.2 Study Characteristics and Quality and Risk of Bias Assessment

A total of 10 articles, all in English, were selected, including one randomized clinical trial (Watts et al. 2019), two retrospective studies (Cheng et al. 2022; Voss et al. 2018), one case control (Seki et al. 2025) and six case series (Bagan et al. 2016; Escobedo et al. 2020; Massaad and Magremanne 2022; Otto et al. 2023; Smith et al. 2022; Tempesta et al. 2022), as shown in the PRISMA flowchart (Figure 1). The studies displayed significant heterogeneity, employing different methodologies, which precluded the possibility of conducting a meta‐analysis. As a result, a narrative synthesis with descriptive analysis was performed, without quantitative evidence for the effects of the interventions. Table 1 reports main characteristics of the studies, that included a total of 8220 patients, the majority of whom were female, with a mean age of 71.45 years (SD ±6.30 years) and a mean follow‐up of 91.3 months. Table 2 includes the summary of the quality and risk of bias assessment analysis, showing that most of the included studies were categorized as having high and moderate methodological quality, with low or moderate risk of bias with only one study with a Low quality and high risk of Bias. A single study (Bagan et al. 2016) was classified as low quality on the basis of the JBI Critical Appraisal Checklist; however, it was decided to keep it in the review because of its clinical relevance and the still little available literature that relevantly addresses the relationship of MRONJ osteoporotic patients undergoing dental implants treated with DEN—a population for which evidence still remains limited. For this reason, all 10 studies were included in data extraction and the final synthesis. See details regarding the JBI Critical Appraisal Checklist in Tables S1 and S2.

**TABLE 1:** Demographic characteristics of patients in the studies included in the systematic review.

Author	Study type	Number of patients	Female	Male	Age			Follow‐up	
					Mean age (years)	Standard deviation (SD)	Range	Time (months)	Lost patients
Seki et al. (2025)	Case control	61	52	9	78.4 (Group 1) 70.9 (Group 2)	± 6.8 (Group 1) ± 8.4 (Group 2)	NR	158.4 ± 76.8 years	NR
Otto et al. (2023)	Case series	16	9	1	70.8	±7.48 years	69.40–67.06* 71.42–67.01**	12	6
Massaad and Magremanne (2022)	Case series	6	4	2	65.6	NR	50–83	NR	0
Smith et al. (2022)	Case series	13	11	2	72	NR	59–87	NR	NR
Cheng et al. (2022)	Retrospective study	3486	3486	0	66.4	±9.10	45–90*	250	NR
Tempesta et al. (2022)	Case series	19	19	0	64.9	±8.3	NR	20	0
Escobedo et al. (2020)	Case series	7	4	3	68	NR	NR	24	0
Watts et al. (2019)	Randomized controlled trials	4550	4550	0	71.9–74.9 (Group 1) 71.8–74.8 (Group 2)	5 (Group 1) 5.1 (Group 2)	60–90 (Total)	84	NR
Voss et al. (2018)	Retrospective study	52	46	6	Female: 76.7 Male: 65.3	Female ±8.2 Male ±14.7	Female: 54–87 Male: 45–80	96	10
Bagan et al. (2016)	Case series	10	10	0	73.7	±11.6	NR	NR	3

*Note:* Group 1: CROSSOVER group; Group 2: LONGTERM group—mean age in 2 different periods (FREEDOM and EXTENSION).

Abbreviation: NR, not reported.

* At time of implant placement.

** Last follow‐up.

**TABLE 2:** Quality and risk of bias assessment.

Study	Study type	Quality assessment tool	Key quality findings	Overall rating
Watts et al. (2019)	RCT	RoB 1	Moderate risk of bias	Moderate quality
Cheng et al. (2022)	Cohort	NOS	8/9 stars	Good quality
Voss et al. (2018)	Cohort	NOS	7/9 stars	Good quality
Seki et al. (2025)	Case control	JBI Checklist	Moderate risk of bias	Moderate quality
Otto et al. (2023)	Case series	JBI Checklist	Moderate risk of bias	Moderate quality
Massaad and Magremanne (2022)	Case series	JBI Checklist	Low risk of bias	Moderate quality
Smith et al. (2022)	Case series	JBI Checklist	Low risk of bias	High quality
Tempesta et al. (2022)	Case series	JBI Checklist	Low risk of bias	Moderate quality
Escobedo et al. (2020)	Case series	JBI Checklist	Low risk of bias	Moderate quality
Bagan et al. (2016)	Case series	JBI Checklist	High risk of bias	Low quality

Abbreviations: JBI Checklist, Joanna Briggs Institute Critical Appraisal Checklist for case series and case control; NOS, Newcastle‐Ottawa Scale for retrospective cohort studies; RCT, randomized controlled trial; RoB 1, ROBINS‐I tool (Cochrane) for randomized controlled trials.

### 3.3 Systemic Conditions and Comorbidities

Although the included studies varied widely in sample size and reporting detail, some provided useful information on patient comorbidities. Chronic conditions such as diabetes, rheumatoid arthritis, hypertension, and chronic kidney disease were mentioned in several studies (Tempesta et al. 2022; Voss et al. 2018; Massaad and Magremanne 2022; Cheng et al. 2022), but their specific impact on implant survival or the risk of MRONJ in patients treated with DEN was not assessed. Further details on patient's systemic conditions are summarized in Table S3. The use of systemic medications, including corticosteroids and anticoagulants, was reported in three studies, but no direct association with MRONJ was explored (Cheng et al. 2022; Massaad and Magremanne 2022; Tempesta et al. 2022). Smoking was reported in four studies (Seki et al. 2025; Otto et al. 2023; Tempesta et al. 2022; Voss et al. 2018); however, because of limited data and the lack of a clear correlation, it is not possible to draw conclusions about its influence on implant outcomes in osteoporotic patients undergoing DEN treatment.

### 3.4 Dental Implants

Patients undergoing implant therapy present a wide range of sample sizes, as shown in Table 3, with studies reporting a total of 1823 implants. The timing of implant placement, which is important in evaluating the potential role of the implant as a trigger for MRONJ, is essential to discriminate between implant presence‐triggered osteonecrosis (IPTO), likely caused by the presence of pre‐existing peri‐implant inflammation in osseointegrated implants, and implant surgery‐triggered osteonecrosis (ISTO), where MRONJ development is directly related to the surgical intervention. Seven studies report the time at which implants were placed, four of which indicate implants placed prior to starting the therapy with DEN (Escobedo et al. 2020; Massaad and Magremanne 2022; Tempesta et al. 2022), whereas three other studies analyze implants positioned after the initiation (Cheng et al. 2022; Otto et al. 2023; Smith et al. 2022). Other studies do not report such details (Bagan et al. 2016; Voss et al. 2018; Watts et al. 2019).

**TABLE 3:** Characteristics of dental implant rehabilitation.

Study	Total N° PTS	Implants								
		Total N°	Denosumab*		IPTO N° PTS (N° IMPL affected/total)	ISTO N° PTS (N° IMPL affected/total)	FAIL N° PTS (N° IMPL)	Antibiotic prophylaxis***	Peri‐implantits	
			N° PTS	N° impl.					Total N° PTS (N° IMPL)	Denosumab* N° PTS (N° IMPL)
Seki et al. (2025)	61	192	3	22	2 (4/7)	—	(13)	NR	8 (35)	NR
Otto et al. (2023)	16	39	3	NR	—	16 (0/39)	0+	Yes	3 (8)	NR
Massaad and Magremanne (2022)	6	43	3	15	6 (17/43)	—	4^++^ (14)	NR	NR	NR
Smith et al. (2022)	13	6	2	6	2 (3/6)	0 (0)	2 (4)	NR	1** (1)	1** (1)
Cheng et al. (2022)	694	1.472	6	29	NR	NR	19 (22)	Yes	NR (8)	NR
Tempesta et al. (2022)	19	37	5	8	19 (37/NR)	0	19 (37)	NR	19 (37)	5 (8)
Escobedo et al. (2020)	7	33	2	4	7 (14/33)	NR	33 (14)	No	NR	NR
Watts et al. (2019)	4550	NR	212	2	0	NR	0	NR	NR	NR
Voss et al. (2018)	52	NR	2	NR	NR	NR	NR	NR	NR	NR
Bagan et al. (2016)	10	1	10	1	NR	NR	NR	NR	NR	NR

*Note:* The column ‘Total N°’ refers to the total number of implants evaluated in each study. Affected = implants with MRONJ. Total = total implants those IPTO patients had.

Abbreviations: N° IMPL, number of implants; N° PTS, number of patients; NR, not reported.

* Includes group of patients in previous bisphosphonates treatment transitioning to denosumab.

** Initially diagnosed as peri‐implantitis, evolved in MRONJ.

*** Antibiotic prophylaxis prior to implant placement.

^+^ Antiresorptive group.

^++^ Not in Denosumab group, IPTO (implant presence‐triggered osteonecrosis), ISTO (implant surgery‐triggered osteonecrosis).

Implant failure, defined as those lost, removed, or associated with MRONJ, is reported in *six studies*, with five occurring in patients undergoing DEN therapy, and a failure rate in these patients ranging from 6% (Cheng et al. 2022) to 100% (Tempesta et al. 2022), including intermediate values of 42.4% (Escobedo et al. 2020) and less than 5% (Smith et al. 2022). One study (Otto et al. 2023) reported no implant failures. The study by Seki et al. reported an implant failure rate of 11.2% for the group treated with antiresorptive therapy (including denosumab) and 2.9% for the control group, assuming PI‐MRONJ cases were already counted among failures (Seki et al. 2025). The use of antibiotic prophylaxis in patients undergoing implant placement is rarely discussed, with only two studies mentioning its use prior to the procedure, and only Otto et al. providing detailed information on the type and administration of the antibiotics (Otto et al. 2023). Briefly, prophylactic systemic antibiotics (amoxicillin/clavulanic acid 875 mg/125 mg or, in case of allergy, clindamycin, 600 mg) were administered 1 day preoperatively and continued for 5–7 days postoperatively. An antiseptic mouthwash of 0.2% chlorhexidine was used immediately before surgery and for 2 weeks after the surgery.

Peri‐implantitis has been reported in some studies like Tempesta et al., whose primary objective was to assess peri‐implant medication‐related osteonecrosis of the jaw (PI‐MRONJ), which provides more comprehensive data on peri‐implant conditions in osteoporotic patients receiving antiresorptive therapy through clinical examinations, probing, and radiographic assessments (Tempesta et al. 2022). In the study by Smith et al., peri‐implantitis was initially diagnosed but was later reclassified as MRONJ (Smith et al. 2022). Massaad et al. reported in their case series 14 implant explantations, which were classified as failures, but none of them belonged to patients using DEN (Massaad and Magremanne 2022). Seki et al. reported peri‐implantitis in patients who initiated antiresorptive therapy after implant placement, with an overall prevalence of 18.2% (35/192 implants) and 13.1% (8/61 patients). However, outcomes were not specified by specific antiresorptives; therefore, the number of denosumab users (or implants) with peri‐implantitis is not reported (Seki et al. 2025).

### 3.5 Denosumab Therapy

From the analysis, two distinct patient groups can be identified: those who transitioned from BP therapy (primarily administered orally) to DEN, and those who received DEN as monotherapy. The data are organized in Table 4, where it is noted that 4 out of the 10 studies present the transition group. Escobedo et al. exclusively reported patients who had previously been treated with BPs before starting DEN (Escobedo et al. 2020). In contrast, two studies presented both groups; however, in the DEN monotherapy group, the patients did not undergo implant therapy (Bagan et al. 2016; Smith et al. 2022). The study by Voss et al., which evaluates transition therapy, included two groups, with only three patients in the monotherapy group. In both groups, the exact number of implants performed was not reported (Voss et al. 2018). Seki et al. compared two groups: a treatment group that initiated antiresorptive therapy (BPs or DEN only after implant placement), and a control group without antiresorptive agents. In both groups, implants had been placed before any antiresorptive use. There was no separate analysis by drug class and no distinct “transition” subgroup. However, within the treatment group, a single case is described with sequential exposure—minodronate followed by DEN—with three implants (Seki et al. 2025).

**TABLE 4:** Characteristics of antiresorptive therapy.

Author	ART osteoporotic									
	Transition BPS‐denosumab						Denosumab monotheraphy			
	N° PTS	N° IMPL.	BPS		Denosumab		N° PTS	N° IMPL	Time (months)	Number of doses
			VIA	Time (months)	Time (months)	Dose*				
Seki et al. (2025)	1	3	Oral	24	24	4*	2	19	84–192	14–32*
Otto et al. (2023)	0	NR	NR	NR	NR	NR	NR	NR	NR	NR
Massaad and Magremanne (2022)	0	0	—	—	—	—	1	5	36	6*
Smith et al. (2022)	4	2	Oral	48–72	> 48	NR	8	0	> 48	NR
Cheng et al. (2022)	NR	NR	NR	NR	NR	NR	6	NR	NR	NR
Tempesta et al. (2022)	0	0	0	—	—	—	5	8	27	4.5*
Escobedo et al. (2020)	2	4	Oral	24–60	18–24	3–4*	0	—	—	—
Watts et al. (2019)	NR	NR	NR	NR	NR	NR	4550	NR	84	NR
Voss et al. (2018)	11	NR	Oral IV Oral +IV	36–120 Average 59 (SD ± 24)	NR	2–9 3.27 (SD ± 2.15)	3	NR	12–24	2–4
Bagan et al. (2016)	9	1	Oral	44.7 ± 25.11	NR	3.4 ± 2.2.	1	0	18	3

*Note:* * Doses were calculated based on the reported duration of denosumab (Prolia) exposure, as they were not directly reported by the studies.

Abbreviations: ADM, route of administration; BPS, bisphosphonates; IMPL, implants; IV, intravenous; NR, not reported; PTS, patients.

The number of doses and duration of DEN therapy reported in many studies were often incomplete or with missing data. The duration of DEN therapy across both groups ranged from 24 to 72 months, with the number of doses varying between 2 to 9 before either the onset of MRONJ or the conclusion of the study. For patients with prior BP therapy, the duration of BP use ranged from 24 to 120 months. It is important to note that four studies (Escobedo et al. 2020; Massaad and Magremanne 2022; Tempesta et al. 2022) did not explicitly report the number of doses administered. However, they did specify the duration of therapy with DEN. Since this medication is typically administered every 6 months, it is possible to estimate the number of doses on the basis of the treatment period provided.

### 3.6 MRONJ

Table 5 reports data on the incidence of MRONJ associated with dental implants in patients who received DEN treatment. Otto et al. did not report any cases of MRONJ (Otto et al. 2023), whereas others, like Cheng et al. and Tempesta et al., documented a significant number of cases, with 22 and 19 patients diagnosed with MRONJ, respectively (Cheng et al. 2022; Tempesta et al. 2022). Regarding the transition between antiresorptive therapies, some patients treated with DEN had previously undergone BP therapy for different time spans, a fact reported in about half of the studies. For example, Smith et al. highlighted patients who had been exposed to BPs for 48 to 72 months before transitioning to DEN, which they used for 18 to 84 months (Smith et al. 2022).

**TABLE 5:** MRONJ characteristics in included studies.

Study	MRONJ OP DEN										
	Total MRONJ N° PTS	Implant trigger									
		N° PTS (N°IMPL)	Stage MRONJ	Transition BPS	SITE (N° IMPL)	DOSE DEN	Exposure ART (months)		Treatment	Resolution	Follow‐up
							BPS	DEN			Time (months)
Seki et al. (2025)	3	3 (5)	II	Yes	MAX (2) MAND (3)	4**	24	24	NR	NR	NR
Otto et al. (2023)	0	0	—	NR	—	—	—	—	—	—	12
Massaad and Magremanne (2022)	1	1 (1)	III	NR	MAND (1)	6**	—	36	Conservative	Stable	NR
Smith et al. (2022)	13	2 (6)	NR	Yes	MAX (6)	3	48–72	12–84	2 Conservative + 1 Surgical	1 healed 1 still being monitored	NR
Cheng et al. (2022)	22	1 (3)	NR	NR	NR	NR	NR	NR	Surgical	All healed	250
Tempesta et al. (2022)	19*	5 (8)*	NR	No	MAND (5)	4.5**	±27	±27	Surgical (all implants removed)	All healed	20
Escobedo et al. (2020)	2	NR (5)	III	Yes	MAX (2) MAND (2)	3–4**	24–60	18–24	Surgical (sequestromy)	1 case Favorable 1 case remains complicated	24
Watts et al. (2019)	13	1 (2)	II	No	MAX (1)	NR	—	60	Surgical	Cured	84
Voss et al. (2018)	52	2 (NR)	II III	Yes	MAX (NR) MAND (NR)	2–9	36–60	15–54	Surgical	Cured	96
Bagan et al. (2016)	10	1 (1)	I	Yes	MAND (1)	2	9	12	Conservative	Not cured	NR

Abbreviations: ART, antiresorptive therapy; BPS, bisphosphonates; MAND, mandibular; MAX, maxilla; N° IMPL, number of implants; N° PTS, number of patients; NR, not reported.

* Peri‐implantitis triggered MRONJ.

** Doses were calculated based on the reported duration of denosumab (Prolia) exposure, as they were not directly provided by the studies.

The table also summarizes the main findings related to osteoporotic patients undergoing DEN therapy in whom implants were considered possible triggers for MRONJ. The dosage of DEN administered before the onset of MRONJ was inconsistently reported across studies. Where reported, patients developed MRONJ after a number of doses ranging from two to nine (Escobedo et al. 2020; Smith et al. 2022; Voss et al. 2018). Instead, Seki et al. required at least 1 year of maintenance after superstructure placement, and cases were defined as patients who initiated antiresorptive therapy (BPs or DEN) after implant placement, with no minimum time interval specified (Seki et al. 2025). Four studies provided exposure time without directly indicating dose, this was calculated as previously done, on the basis of the 6‐month interval between subcutaneous injections of DEN. Considering patients transitioning from BPs to DEN, the duration of exposure to both drugs before MRONJ development varied significantly among the studies. In Smith et al., patients were exposed to DEN for 18 to 84 months, and BPs for 48 to 72 months prior to MRONJ onset (Smith et al. 2022). Escobedo et al. reported that DEN exposure lasted between 18 and 24 months, whereas BP exposure ranged from 24 to 60 months (Escobedo et al. 2020). In the study by Voss et al., the exposure to DEN was between 15 and 54 months, whereas BP exposure spanned 36 to 60 months (Voss et al. 2018). For other studies, exposure data were not reported (Cheng et al. 2022; Otto et al. 2023).

Only three studies provided detailed information regarding MRONJ stages at diagnosis. Escobedo et al. reported that patients were diagnosed with MRONJ stage III (Escobedo et al. 2020), whereas Watts et al. and Voss et al. reported MRONJ stages II and III, indicating varying degrees of severity across the studies. Notably, the only patient classified as stage III was osteoporotic, treated with DEN (Voss et al. 2018; Watts et al. 2019). In Seki et al., only one case of MRONJ had a reported stage from a single patient who transitioned from a BP to DEN and was described as peri‐implant MRONJ stage 2; the other cases of MRONJ were reported without staging (Seki et al. 2025). The location of MRONJ lesions (in the maxilla or mandible) was detailed in some studies, with an overall similar distribution between the two areas. However, some studies highlighted a predominance of lesions in one region, such as Smith et al., where six lesions were reported in the mandible (Smith et al. 2022), and Tempesta et al., where five cases were found in the maxilla (Tempesta et al. 2022). In the study by Massaad et al., 17 of the 43 implants triggered the development of MRONJ (Massaad and Magremanne 2022).

Treatment approaches varied, with some studies reporting the use of surgical interventions, whereas others opted for conservative management. Two studies reported favorable outcomes in patients who underwent surgical treatment (Cheng et al. 2022; Tempesta et al. 2022). Smith et al. reported that one of the cases with complications occurred in a patient who developed breast cancer during treatment (Smith et al. 2022). In contrast, Escobedo et al. described mixed results, where one surgically treated case showed favorable outcomes, but the other remained complicated (Escobedo et al. 2020). Conservative treatments generally resulted in less favorable outcomes. The follow‐up periods varied greatly across the studies, ranging from 12 months to 250 months. Cheng et al. documented the longest follow‐up period, with 250 months of patient observation (Cheng et al. 2022).

### 3.7 Descriptive Analysis of Summated Data

Table 6 reports summarized data, providing a comparison of the number of patients across the different conditions investigated and undergoing implant therapy, compared with the general population and the total number of patients with MRONJ. Across all studies, a total of 5428 patients were examined, of whom 5043 were diagnosed with osteoporosis. A total of 1817 dental implants were analyzed across the studies, with Cheng et al. contributing the majority (1472 implants) (Cheng et al. 2022), whereas Bagan et al. reported only a single implant case (Bagan et al. 2016). Of the total patient population, 366 were undergoing treatment with DEN, with marked differences across the included studies.

**TABLE 6:** Summary of patients, implants, and MRONJ cases in osteoporotic individuals treated with denosumab.

Study	Patients				Implants (N°)		MRONJ cases in OP patients on DEN with implants	
	N° total patients	N° OP	N° DEN	N° total MRONJ	Total	DEN	N° patients	N° implants
Seki et al. (2025)	61	61	3	3	192	22	1	1*
Otto et al. (2023)	16	11	3	0	33	NR	0	0
Massaad and Magremanne (2022)	6	4	1	6	43	15	1	1
Smith et al. (2022)	13	11	2	13	6	6	2	6
Cheng et al. (2022)	694	323	114	22	1472	417	3	1
Tempesta et al. (2022)	19	19	5	19	37	37	5	8*
Escobedo et al. (2020)	7	2	2	2	33	4	2	4
Watts et al. (2019)	4550	4550	212	13	NR	NR	1	2
Voss et al. (2018)	52	52	14	52	NR	NR	2	NR
Bagan et al. (2016)	10	10	10	10	1	1	1	1
TOTAL	5428	5.043	366	140	1.817	502	18	24

*Note:* Gray rows indicate higher‐quality study designs (e.g., cohort studies, RCT). MRONJ cases in OP patients on DEN with implants: number of MRONJ cases reported specifically in osteoporotic patients under denosumab therapy, with dental implants identified as a potential triggering factor.

Abbreviations: DEN, denosumab; IMPL, implants; N°, number; NR, not reported; OP, osteoporosis.

* Associated with peri‐implantitis.

Overall, 140 cases of MRONJ were reported across all studies. Among these, 18 patients developed MRONJ associated with DEN and dental implants, which represents the target group of this study. Considering the 140 patients with MRONJ, the number of implants associated with MRONJ in patients treated with DEN was 24. Implants were identified as a trigger for MRONJ in 8 of the studies (Bagan et al. 2016; Cheng et al. 2022; Escobedo et al. 2020; Massaad and Magremanne 2022; Smith et al. 2022; Tempesta et al. 2022; Voss et al. 2018), whereas in one, there were no cases of MRONJ triggered by dental implants (Otto et al. 2023), and in the last, the data were not reported (Watts et al. 2019).

Concerning the incidence of MRONJ associated with implant placement timing (IPTO/ISTO), most of the included articles analyzed only one type of implant timing, either IPTO or ISTO, without direct comparisons between these two groups, and only two studies (Escobedo et al. 2020; Massaad and Magremanne 2022) directly refer to the IPTO/ISTO classification. Our study revealed more data in the IPTO groups compared to the ISTO groups, as shown in Table 3. Although such data must be interpreted with caution because of the significant heterogeneity among studies, a careful evaluation of the articles allows for meaningful analyses. Seki et al. (2025) and Massaad and Magremanne 2022 highlight that all cases belonged to the IPTO group, which is considered a potentially higher‐risk group for MRONJ according to the literature (Massaad and Magremanne 2022) and Escobedo et al. (2020) confirms the association between PI‐MRONJ cases and previously osseointegrated implants (Escobedo et al. 2020). Important to note that the analysis of MRONJ across studies (Bagan et al. 2016; Cheng et al. 2022; Escobedo et al. 2020; Massaad and Magremanne 2022; Smith et al. 2022; Tempesta et al. 2022; Voss et al. 2018), highlights that the observed PI‐MRONJ cases are consistent with an implant‐presence–triggered (IPTO) pattern. Conversely, studies involving ISTO (Otto et al. 2023) implants did not assess MRONJ, preventing risk estimation.

When evaluating the influence of transition therapy from BPs to DEN on the increased risk of MRONJ, it is noted that most of the articles that included transition therapy did not evaluate the increased risk for MRONJ. The study by Voss et al. (2018) is the only one that describes a higher rate of MRONJ recurrence in this type of therapy in osteoporotic patients for DEN.

These results demonstrate significant variability in the prevalence of MRONJ across different studies, likely influenced by factors such as sample size, duration of follow‐up, patient demographics, and study methodologies. Therefore, these findings should be interpreted with caution, taking into account the inherent limitations of each study.

## 4 Discussion

This systematic review analyzed the impact of dental implants in patients with osteoporosis undergoing treatment with DEN and the development of MRONJ. The available data were collected from multiple recent studies, emphasizing variations in the incidence of MRONJ and the relationship between DEN use and implant placement. The reviewed studies revealed that among the 5428 patients treated with DEN, a significant proportion presented cases of MRONJ. There was one randomized trial presenting “some concerns,” which leads us to one of the primary challenges encountered in this review: that was the significant heterogeneity across studies, both in terms of patient demographics and the timing of interventions related to antiresorptive therapy. Regarding the quality of the studies, most were classified as moderate and good/high quality, with one of the included case series (Bagan et al. 2016) classified as low quality. Nonetheless, we chose to maintain it because of the lack of high‐level evidence in this population, and it was interpreted cautiously, considering its small sample size. Despite these limitations, some consistent trends can be observed that deserve further discussion.

The literature presents numerous studies evaluating the occurrence of MRONJ in osteoporotic patients undergoing antiresorptive therapy. According to Khan et al., the occurrence of MRONJ in patients using low doses of antiresorptives is low, although there are limitations in the accurate assessment of its incidence (Khan et al. 2015). In light of this, the study by Watts et al. has been a key reference regarding the incidence of MRONJ in osteoporotic patients, reporting an overall incidence rate of 5.2 per 10,000 patients per year (Watts et al. 2019). In a more recent study, the incidence of MRONJ following DEN administration was 4.1%, with no statistically significant factors influencing the incidence among patients receiving DEN treatment (Jung et al. 2022). Furthermore, according to studies, the risk of MRONJ increases with the duration of exposure to DEN, with rates of 0.04% at 3 years, 0.06% at 5 years, and 0.44% at 10 years of treatment (Nicolatou‐Galitis et al. 2019). In our study, among the 366 patients who underwent implant procedures, 18 developed MRONJ, involving 24 implants in total. However, it is important to interpret these figures with caution because of the variability and limitations in the reviewed studies.

The existence of associated comorbidities has been suggested as possible risk factors for the development and prognosis of MRONJ; however, there is still no consensual data to validate this position, mainly because of the inconsistent manner in which these conditions have been reported in the literature (Bedogni et al. 2024; Ruggiero et al. 2022; Schimmel et al. 2017; Stavropoulos et al. 2018). In this review, four studies reported comorbidities, the most common being diabetes, hypertension, and rheumatoid arthritis. In particular, one study observed an association between diabetes and reduced implant survival rates (Cheng et al. 2022). It is important to note that diabetes impacts the ratio of receptor activator of nuclear factor kappa‐B ligand (RANKL) to osteoprotegerin (OPG), and during hyperglycemia, this imbalance can promote increased bone resorption; additionally, these patients are more susceptible to both systemic and localized infections, which can negatively affect osseointegration (Aghaloo et al. 2019). However, the exact role of these comorbidities in contributing to the development of MRONJ has not yet been clearly elucidated.

As outlined in Briot et al. and the Guidelines on the management of osteoporosis and fragility fractures (2018), the use of corticosteroids is already established as a risk factor, as it can induce secondary osteoporosis (Briot and Roux 2015; SIGG et al. 2018), even with low doses or during short treatment periods of less than 30 days (Hsu et al. 2024). It is known that corticosteroids can be a risk factor for the development of aseptic osteonecrosis by increasing bone resorption and suppressing the ossification process, in addition to raising the risk of infections (Shibahara 2019). In our study, only Cheng et al. (2022) and Massaad and Magremanne (2022) reported corticosteroid use; however, Cheng et al. (2022) provided no further details, and although use differed significantly between the analyzed subgroups, no association with implant failure was found (Cheng et al. 2022). Furthermore, the use of anticoagulants and antihypertensives can complicate the surgical management of dental implants and invasive oral procedures (Aghaloo et al. 2019). Therefore, it is crucial to consider polypharmacy and comorbidities when planning dental treatments for these patients, emphasizing the importance of a multidisciplinary approach to mitigate MRONJ risks and ensure long‐term therapeutic success (Ruggiero et al. 2022).

The role of smoking in the development of MRONJ has been established as one of the risk factors (Bedogni et al. 2024; Ruggiero et al. 2022). In this review, the relationship between smoking and MRONJ was reported in a limited manner, with few studies directly mentioning the impact of smoking. Although specific data on smoking were scarce in the analyzed studies, the medical literature suggests that smoking can lead to complications in oral surgeries and can impair both bone and soft tissue healing, contributing to implant failure and peri‐implantitis, potentially increasing the risk of developing MRONJ (Bedogni et al. 2024; Landi et al. 2024; Ruggiero et al. 2022). Evaluating risk factors for dental implants, Chen et al. (2013) concluded that individuals who smoke are more likely to experience dental implant failure (Chen et al. 2013). On the other hand, Kim et al. (2020) reported that smoking was not a significant factor in their study (Kim et al. 2020). Aljohani et al. (2017), when evaluating the relationship between smoking and MRONJ, found that only 5.5% of the patients were smokers, which did not stand out as a relevant risk factor (Aljohani et al. 2017). Therefore, despite the limited discussion of smoking in the articles reviewed, smoking is considered an additional risk factor for patients undergoing antiresorptive therapy and dental implant procedures.

In terms of DEN use, we found that few studies address its use in osteoporotic patients in relation to dental implants and the impact on MRONJ development. Most of the studies, predominantly case series, often report only one patient with scarce information. In the included studies, a lack of standardization and the absence of important data, such as duration of exposure and number of doses before the onset of MRONJ, were observed. The SIPMO‐SICMF 2024, MASCC/ISOO, and AAOMS guidelines and recommendations updates highlight that agent type, dosing schedule, treatment duration, and cumulative dose are relevant factors in assessing medication‐related risks (Bedogni et al. 2024; Ruggiero et al. 2022; Yarom et al. 2019). Hoefert et al. 2017, in a study on jaw osteonecrosis associated with DEN, reported an average of 14 doses before the onset of MRONJ, although only one osteoporotic patient was included (Hoefert et al. 2017). In our review, a few studies provided clear data. Providing such information in a more detailed and consistent manner is important, particularly for assessing cumulative doses and determining whether there is a minimum number of doses or duration of exposure before MRONJ develops. Treatment duration and cumulative dose of DEN may influence outcomes; in Voss et al. (2018), the median DEN exposure was 59 months and was discussed as potentially associated with a higher risk of MRONJ, whereas Smith et al. (2022) documented DEN exposure of approximately 12 to 84 months in their implant‐associated cases; however, Seki et al. (2025), reported 84 to 192 months of exposure in 20 patients with only three cases of MRONJ/PI‐MRONJ, and the single denosumab case had a shorter exposure than the other two DEN‐treated patients in the cohort, indicating that longer exposure does not necessarily imply a higher risk. Given these contrasting findings, it is important to continue close monitoring of patients undergoing long‐term treatment who receive dental implants, especially considering the potential implications of MRONJ on quality of life and overall oral health.

Another important aspect found in our study concerns the comparative risks of MRONJ in patients treated with DEN alone versus those who transitioned from BP therapy to DEN. The data suggest that patients with a history of BP use prior to transitioning to DEN may be at risk of developing MRONJ more frequently compared with those on DEN monotherapy, attributed to the potentially life‐long persistence of BPs in the bone after administration (Voss et al. 2018), that can continue to influence bone remodeling long after discontinuation (Baron et al. 2011). When combined with DEN's potent inhibition of osteoclast activity, this may increase bone mineral density and further reduce bone turnover (Kendler et al. 2022). Smith et al. and Escobedo et al. (2020) reported that patients who received BPs for prolonged periods (48–72 months) prior to initiating DEN had a higher incidence of MRONJ (Escobedo et al. 2020; Smith et al. 2022). The study by Seki et al. (2025), although it did not include a transition subgroup and had only three patients on DEN therapy in the antiresorptive group, of the three cases of MRONJ identified, the only one that occurred under DEN therapy was the patient who transitioned from BPs to DEN (Seki et al. 2025). In contrast, patients who received DEN monotherapy for shorter periods had a lower incidence of MRONJ. The study by Voss et al. 2018, which addresses this issue, found that patients transitioning from BPs to DEN had higher recurrence rates after surgical treatment compared to those receiving BPs or DEN monotherapy (Voss et al. 2018). Consistently, previous studies have noted a higher prevalence of MRONJ in patients transitioning from BPs to DEN compared to those receiving either BPs or DEN alone, particularly in higher‐risk cancer patients, highlighting a trend toward earlier development of MRONJ in the transition therapy group (Loyson et al. 2018; Srivastava et al. 2021).

Considering the data of the studies included in this review, the overall incidence of MRONJ in patients undergoing dental implant placement while on DEN therapy was relatively low (18 cases out of 366 patients). This suggests that, although there is an increased risk of MRONJ, it remains a rare complication in DEN‐treated osteoporotic patients, consistent with findings in previous literature (Adler et al. 2016; Khan et al. 2015; Ruggiero et al. 2022). However, the variability in the number of implants and patient follow‐up times makes it difficult to draw firm conclusions on the true risk, especially considering that most studies did not differentiate between implants placed before, during, or after DEN administration.

A significant aspect of this review involves the distinction between Implant Presence‐Triggered Osteonecrosis (IPTO) and Implant Surgery‐Triggered Osteonecrosis (ISTO). This classification has been studied in the literature to determine whether the timing of implant placement in relation to the onset of antiresorptive therapy plays a crucial role in the risk of MRONJ development (Giovannacci et al. 2019). Holzinger et al. reported a higher risk of developing MRONJ in patients who underwent implant placement after starting antiresorptive therapy (Holzinger et al. 2014). Meanwhile, Escobedo et al., in their literature review, found a higher incidence of MRONJ in patients with osseointegrated implants for at least 1 year (Escobedo et al. 2020). However, it is important to note that these studies primarily focus on BP therapy. Massaad and Magremanne investigated a group of IPTO patients and highlighted the occurrence of MRONJ linked to IPTO despite the limitations of their study (Massaad and Magremanne 2022). This suggests that DEN therapy may act as a direct trigger for osteonecrosis. In our review, the association between IPTO and a higher risk of MRONJ is difficult to establish because of the heterogeneity of the studies. Seki et al. (2025) did not use the IPTO/ISTO terminology; however, they required at least 1 year of maintenance after superstructure placement and defined cases as patients who initiated antiresorptive therapy (BPs or DEN) after implant placement. The observed PI‐MRONJ events arose around functioning implants during maintenance, which is consistent with an IPTO pattern (Seki et al. 2025).

These data reinforce the importance of careful consideration regarding the timing of dental implant surgery, as well as properly advising and monitoring patients with pre‐existing osseointegrated implants who need to initiate therapy with antiresorptive medications (Granate‐Marques et al. 2019).

Anatomical and radiological variables may also contribute to the development of MRONJ in patients with dental implants. Studies that evaluated implant success and failure in patients undergoing treatment with antiresorptive agents, including DEN, indicate that posterior regions of the mandible may be more susceptible to MRONJ because of biomechanical stress and vascular impairment (Kim et al. 2020; Granate‐Marques et al. 2019; Giovannacci et al. 2016). The possibility of procedures such as grafts being an additional risk factor is suggested, but still little reported in the literature, with little evidence (Kim et al. 2020; Granate‐Marques et al. 2019). Pre‐existing marginal bone loss and radiographic signs of osteosclerosis have been discussed as potential predisposing factors (Kim et al. 2020). Although radiographic findings are not a consensus and should be associated with a clinical picture, guidelines recommend, in addition to traditional and panoramic radiographs, the use of cone beam computed tomography or multidetector computed tomography to detect alterations such as sclerosis, osteolysis, and osteolytic changes early and assist in surgical planning (Bedogni et al. 2024; Khan et al. 2015). These characteristics, sometimes observed in implant areas before the onset of symptoms, may reflect a prodromal stage of MRONJ (Giovannacci et al. 2016; Granate‐Marques et al. 2019). In our review, some studies reported a higher frequency of MRONJ in implants located in the posterior mandibular and maxillary regions (Seki et al. 2025; Massaad and Magremanne 2022; Tempesta et al. 2022), suggesting that these areas may be more susceptible to biomechanical overload and reduced vascularization. Only one study (Otto et al. 2023) provided specific information on bone volume prior to implant placement. Furthermore, radiographic evidence of osteosclerotic changes prior to implant insertion was observed in some cases (Voss et al. 2018; Massaad and Magremanne 2022), raising the question of whether such findings could represent predisposing factors. However, these aspects have been reported inconsistently and superficially, and no study has addressed them systematically—highlighting a significant gap in the current literature and a possible direction for future research. Although none of the studies included in our systematic review addressed these aspects systematically, their sporadic mention in some case series emphasizes the need for future investigations to evaluate anatomical location, pre‐existing radiological features, and bone quality prior to implant placement as potential risk indicators for MRONJ.

The success or failure of dental implants and their potential influence on MRONJ development represent another important theme, together with the role of peri‐implantitis. Implant failure, defined as the loss or removal of implants or the presence of implants involved in MRONJ, was reported in several studies. Stavropoulos et al. 2018, considered that the implant presence (IPTO) was the trigger for MRONJ in about 30% of the patients, whereas in about 16% of the cases, the lesion was related to implant installation or explantation (Stavropoulos et al. 2018). Although implant placement in osteoporotic patients on DEN therapy is not inherently contraindicated, the review suggests that implant failure may increase the risk of MRONJ. Specifically, implants that fail because of peri‐implantitis or infection could act as local triggers for the onset of osteonecrosis; this highlights the importance of close monitoring of implant sites, particularly in patients with pre‐existing risk factors, such as prolonged BP use prior to transitioning to DEN (Bedogni et al. 2024; Escobedo et al. 2020). In addition, several studies suggest peri‐implant inflammation and infection may act as triggers for osteonecrosis, particularly in patients receiving antiresorptive therapies (Bedogni et al. 2024; Holzinger et al. 2014; Kuroshima et al. 2022; Li and Leung 2024; Otto et al. 2023; Pichardo et al. 2020; Ruggiero et al. 2022). Few studies described with major details peri‐implantitis triggered MRONJ and reported a form of peri‐implantitis where, after removal, the implants remained integrated into the underlying bone (Pogrel and Ruggiero 2018), considered an early form of MRONJ characterized by bone necrosis, extensive osteolysis and block‐type bone sequestration where the implant remains integrated with the bone (Tempesta et al. 2022). However, the limited number of studies evaluating peri‐implantitis in this context highlights the need for more focused research on the relationship between peri‐implant disease and MRONJ development in DEN patients.

In general, the incidence of MRONJ in patients using low doses of antiresorptive medications in osteoporosis patients remains low (Bedogni et al. 2024; Khan et al. 2015; Ruggiero et al. 2022). In our review, the studies seem to suggest confirmation of this trend, which should be analyzed with great caution because of the heterogeneous nature of the included studies and considering that MRONJ appears as a relatively rare complication in this subgroup of patients. The data further indicate that the incidence of MRONJ in patients on DEN therapy, particularly following implant procedures, is low but significant enough to warrant caution. The variation in data regarding exposure times, ranging from 12 to 192 months, suggests that there is a risk of MRONJ even with relatively short exposure periods. The trigger for MRONJ appears to be dental implants in a number of cases, emphasizing the need for careful dental management and monitoring in patients receiving DEN (Smith et al. 2022; Tempesta et al. 2022; Voss et al. 2018).

The majority of patients with MRONJ were treated surgically, as surgical treatment was more frequently associated with resolution of the condition, whereas conservative management often led to prolonged or unresolved cases. Recent studies have shown that surgery, when performed appropriately and in a timely manner, can lead to a resolution of the condition, significantly improving the patient's quality of life (Bedogni et al. 2024; Ruggiero et al. 2022). Our review also supports the evidence that surgical management of MRONJ, particularly in advanced stages, yields better outcomes compared to conservative treatments. Studies such as those by Cheng et al. and Tempesta et al. demonstrated that surgical debridement or partial maxillectomy, followed by bone contouring and soft‐tissue closure, provided favorable outcomes in most patients (Cheng et al. 2022; Tempesta et al. 2022).

As with many studies published on this topic, this systematic review presents several limitations. The high heterogeneity and lack of important data and variability made it difficult to standardize the results, which ultimately precluded the possibility of conducting a meta‐analysis. Most of the included articles were case series, one of which was of low quality and had a higher risk of bias, and only two were retrospective cohort studies of good quality, one case control and one randomized trial of moderate quality. The total number of studies that met the inclusion criteria was limited, with only 10 studies included in the final analysis, and some of these studies had very small sample sizes, which reflects the current state of the literature on the subject. Another limitation of our study is the possibility of missing some articles, despite the extensive search, because of the use of different nomenclatures, language restrictions and the exclusion of some databases. The lack of standardization across the studies is also another significant limitation for comparing the results. For example, there were inconsistencies in distinguishing between different patient groups and in reporting essential data points, such as the number of dental implants performed, the timing of implant placement relative to antiresorptive therapy, or the number of drug doses, duration of exposure, and length of follow‐up. Another significant limitation of this review is the fact that patients with DEN monotherapy were often not distinguished from patients transitioning from BPs to DEN, limiting the interpretation of results and fostering the need to perform future studies capable of isolating these effects through multivariate analysis. All of this leads us to pursue a cautious analysis of the data, describing and highlighting possible factors that should be investigated in the future with more robust studies, such as randomized clinical trials, and that may provide stronger evidence on the topic and support more reliable conclusions to guide treatment planning and both clinical and surgical decision‐making.

## 5 Conclusions

The results of this review highlight that osteoporotic patients, particularly postmenopausal women who have initiated or are due to initiate antiresorptive therapy and who require dental implant procedures, must be considered at risk of developing MRONJ. Although the literature indicates a low incidence, it remains crucial to assess the risk of this condition and its impact on patients' quality of life. However, this systematic review presents several limitations that must be acknowledged. The heterogeneity of the included studies, the predominance of case series, and the absence of randomized controlled trials significantly limit the generalizability and strength of the conclusions. Moreover, inconsistencies in diagnostic criteria, follow‐up duration, and reporting of antiresorptive therapy regimens hinder direct comparison between studies. The small sample sizes and retrospective designs further restrict the ability to establish causal relationships or to assess the long‐term effects of treatment modalities. Despite these constraints, this review highlights clinically relevant findings, including the influence of transition therapy from BPs to DEN, the potential role of peri‐implantitis in implant‐related MRONJ, and the favorable outcomes of surgical management compared with conservative approaches. Future research should prioritize well‐designed, multicenter prospective trials with standardized diagnostic protocols, homogeneous patient selection, and long‐term follow‐up to generate more robust evidence. Strengthening methodological quality will be crucial to refining clinical guidelines, optimizing risk assessment, and ultimately improving prevention and management strategies for MRONJ in patients at risk.

## Author Contributions

**Rebheca Maria Pereira Santos:** conceptualization, investigation, writing – original draft. **Giulia Ottaviani:** conceptualization, writing – review and editing, methodology. **Roberto Di Lenarda:** supervision, writing – review and editing, validation. **Matteo Biasotto:** conceptualization, supervision, data curation, writing – review and editing. **Katia Rupel:** conceptualization, supervision, investigation, writing – original draft, methodology, formal analysis.

## Funding

The authors have nothing to report.

## Ethics Statement

The authors have nothing to report.

## Consent

The authors have nothing to report.

## Conflicts of Interest

The authors declare no conflicts of interest.

## Supporting information


**Table S1:** Joanna Briggs Institute (JBI) Critical Appraisal Checklist for assessment of risk of bias in case series papers.

**Table S2:** Joanna Briggs Institute (JBI) Critical Appraisal Checklist for assessment of risk of bias in case series papers.

**Table S3:** Systemic conditions and comorbidities. Pts (patients), NR (not reported), impl (dental implants), BPs + DEN (patients with previous bisphosphonate treatment), DEN (patients only in denosumab treatment), DBT (diabetes), ATF (atrial fibrillation), HTN (hypertension), HTD (hypothyroidism), GER (gastroesophageal reflux), GCT (glucocorticoids), RMA (rheumatoid arthritis), BTB (beta‐blockers), STA (steroid‐application) ATC (anticoagulant), CKD (chronic kidney disease), SPA (Ankylosing spondylitis).

## Data Availability

All data are available upon direct request to the corresponding author.
