# Supplementary material for: MRONJ Risk Related to Dental Implants in Osteoporosis Treated With Denosumab: A Systematic Review
Source: Oral Dis. 2026 Jan 8;32(4):910–25. doi: 10.1111/odi.70181 (PMC13248576; doi:10.1111/odi.70181)
Supplement: Supplementary file 1 — Table S1: Joanna Briggs Institute (JBI) Critical Appraisal Checklist for assessment of risk of bias in case series papers. [file ODI-32-910-s001.docx]

| **Critical Appraisal tools for use in JBI Systematic Reviews** | | | | | | |
| --- | --- | --- | --- | --- | --- | --- |
|  | Otto et al 2023 | Smith et al 2022 | Massaad et al 2022 | Tempesta et al 2021 | Escobedo et al 2020 | Bagan et al 2016 |
| 1. Were there clear criteria for inclusion in the case series? | Yes, | Yes | Yes | Yes | Yes | Yes |
| 2. Was the condition measured in a standard reliable way for all participants included in the case series? | Yes | Yes | Yes | Yes | Yes | Yes |
| 3. Were valid methods used for identification of the condition for all participants included in the case series? | Yes | Yes | Yes | Yes | Yes | Yes |
| 4. Did the case series have consecutive inclusion of participants? | Yes | Yes | Unclear | Unclear | Yes | Unclear |
| 5. Did the case series have complete inclusion of participants? | Yes | Yes | Yes | Yes | Yes | Yes |
| 6. Was there clear reporting of the demographics of the participants in the study? | Yes | Yes | Yes | Yes | Yes | Yes |
| 7. Was there clear reporting of clinical information of the participants? | Yes | Yes | Yes | Yes | Yes | Yes |
| 8. Were the outcomes or follow-up results of cases clearly reported? | Yes | Yes | Yes | Yes | Yes | Yes |
| 1. Was there clear reporting of the presenting site(s)/clinic(s) demographic information? | No | Yes | No | Yes | Unclear | No |
| 10. Was statistical analysis appropriate? (“In case series, descriptive statistics such as frequencies, percentages, means, and standard deviations are considered appropriate for summarizing patient demographics, interventions and outcomes.”) | Yes | Yes | Yes | Yes | Yes | Yes |
| Overall appraisal: | Moderate Quality / Moderate Risk of Bias | High Quality / Low Risk of Bias | Moderate Quality / Moderate Risk of Bia | Moderate Quality / Moderate Risk of Bia | Moderate Quality / Moderate Risk of Bias | Low Quality / High Risk of Bias |

**Supplementary Table 1.** Joanna Briggs Institute (JBI) Critical Appraisal Checklist for assessment of risk of bias in case series papers.
