# Supplementary material for: MRONJ Risk Related to Dental Implants in Osteoporosis Treated With Denosumab: A Systematic Review
Source: Oral Dis. 2026 Jan 8;32(4):910–25. doi: 10.1111/odi.70181 (PMC13248576; doi:10.1111/odi.70181)
Supplement: Supplementary file 2 — Table S2: Joanna Briggs Institute (JBI) Critical Appraisal Checklist for assessment of risk of bias in case series papers. [file ODI-32-910-s002.docx]

| **Critical Appraisal tools for use in JBI Systematic Reviews** | |
| --- | --- |
| **Question** | **Response** |
| 1. Were the groups comparable other than the presence/absence of disease? | **No** |
| 2. Were cases and controls matched appropriately? | **Unclear** |
| 3. Were the same criteria used for identification of cases and controls? | **Yes** |
| 4. Was exposure measured in a standard, valid and reliable way? | **Yes** |
| 5. Was exposure measured the same way for cases and controls? | **Yes** |
| 6. Were confounding factors identified? | **Yes** |
| 7. Were strategies to deal with confounding stated? | **Yes** |
| 8. Were outcomes assessed in a standard, valid and reliable way for both groups? | **Yes** |
| 1. Was the exposure period long enough to be meaningful? | **Yes** |
| 10. Was appropriate statistical analysis used? | **Yes** |
| **Overall appraisal** | **Moderate Quality / Moderate Risk of Bias** |

**Supplementary Table 2.** Joanna Briggs Institute (JBI) Critical Appraisal Checklist for assessment of risk of bias in case series papers.
