# Supplementary material for: MRONJ Risk Related to Dental Implants in Osteoporosis Treated With Denosumab: A Systematic Review
Source: Oral Dis. 2026 Jan 8;32(4):910–25. doi: 10.1111/odi.70181 (PMC13248576; doi:10.1111/odi.70181)
Supplement: Supplementary file 3 — Table S3: Systemic conditions and comorbidities. Pts (patients), NR (not reported), impl (dental implants), BPs + DEN (patients with previous bisphosphonate treatment), DEN (patients only in denosumab treatment), DBT (diabetes), ATF (atrial fibrillation), HTN (hypertension), HTD (hypothyroidism), GER (gastroesophageal reflux), GCT (glucocorticoids), RMA (rheumatoid arthritis), BTB (beta‐blockers), STA (steroid‐application) ATC (anticoagulant), CKD (chronic kidney disease), SPA (Ankylosing spondylitis). [file ODI-32-910-s003.docx]

| AUTHOR | OSTEOPOROSIS | | COMORBIDITIES | | SMOKING  IN DEN  GROUP | OTHER MEDICATIONS  IN DEN  GROUP |
| --- | --- | --- | --- | --- | --- | --- |
|  | TOTAL  n° pts | DEN + IMPL  n° pts | ALL GROUPS  n° pts | DEN+OSTEO+IMPL GROUP  n° pts |  |  |
| Seki et al.2025 | 61 | 3 | NR | NR | 3 | NR |
| Otto et al. 2023 | 11 | NR | NR | NR | 1 | NR |
| Massaad et al. 2022 | 4 | 1 | 3 HTN  1 CKD  1 SPA | 1 HTN | 0 | 1 GCT |
| Smith et al. 2022 | 11 | 2 pts | NR | NR | NR | NR |
| Cheng et al. 2022 | 323 | 6 | DBT (data NR) | NR | YES (data NR) | GCT (data NR) |
| Tempesta et al. 2022 | 19 | 5 | 3 DBT  1 ATF  2 HTN 2 HTD 1 GER | 2 DBT | 2 | 2 ATC |
| Escobedo et al. 2020 | 2 (4 impl) | 2 (4 impl) | NR | - | NR | NR |
| Watts et al. 2020 | 4550 | 212 | NR | NR | 0 | NR |
| Voss et al. 2018 | 52 | 2 | 8 DBT 9 RMA | BPs+DEN - 1 DBT, 2 RMA  DEN - 1 DBT, 1 RMA | 1 BPs+DEN  1 DEN | BPs+DEN - 2 ATC  DEN - 1 ATC |
| Bagan et al. 2016 | 10 | 1 | NR | NR | NR | NR |

**Supplementary Table 3. Systemic conditions and comorbidities**. Pts (patients), NR (not reported), impl (dental implants), BPs+DEN (patients with previous bisphosphonate treatment), DEN (patients only in denosumab treatment), DBT (diabetes), ATF (atrial fibrillation), HTN (hypertension), HTD (hypothyroidism), GER (gastroesophageal reflux), GCT (glucocorticoids), RMA (rheumatoid arthritis), BTB (beta-blockers), STA (steroid-application) ATC (anticoagulant), CKD (chronic kidney disease), SPA (Ankylosing spondylitis).
